# Identification of a novel linear epitope on the NS1 protein of avian influenza virus

**DOI:** 10.1186/s12866-015-0507-4

**Published:** 2015-08-20

**Authors:** Xuexia Wen, Jiashan Sun, Xiurong Wang, Hongmei Bao, Yuhui Zhao, Xianying Zeng, Xiaolong Xu, Yong Ma, Linlin Gu, Hualan Chen

**Affiliations:** Animal Influenza Laboratory of the Ministry of Agriculture and State Key Laboratory of Veterinary Biotechnology, Harbin Veterinary Research Institute, Chinese Academy of Agricultural Sciences, Harbin, People’s Republic of China; College of Veterinary Medicine, China Agricultural University, Beijing, People’s Republic of China

## Abstract

**Background:**

The NS1 protein of avian influenza virus (AIV) is an important virulent factor of AIV. It has been shown to counteract host type I interferon response, to mediate host cell apoptosis, and to regulate the process of protein synthesis. The identification of AIV epitopes on NS1 protein is important for understanding influenza virus pathogenesis.

**Results:**

In this paper, we describe the generation, identification, and epitope mapping of a NS1 protein-specific monoclonal antibody (MAb) D9. First, to induce the production of MAbs, BALB/c mice were immunized with a purified recombinant NS1 expressed in *E. coli*. The spleen cells from the immunized mice were fused with myeloma cells SP2/0, and through screening via indirect ELISAs, a MAb, named D9, was identified. Western blot assay results showed that MAb D9 reacted strongly with the recombinant NS1. Confocal laser scanning microscopy showed that this MAb also reacts with NS1 expressed in 293T cells that had been transfected with eukaryotic recombinant plasmids. Results from screening a phage display random 7-mer peptide library with MAb D9 demonstrated that it recognizes phages displaying peptides with the consensus peptide WNLNTV--VS, which closely matches the ^182^WNDNTVRVS^190^ of AIV NS1. Further identification of the displayed epitope was performed with a set of truncated polypeptides expressed as glutathione S-transferase fusion proteins, and the motif ^182^WNDNT^186^ was defined as the minimal unit of the linear B cell epitope recognized by MAb D9 in western blot assays. Moreover, homology analysis showed that this epitope is a conserved motif among AIV.

**Conclusions:**

We identified a conserved linear epitope, WNDNT, on the AIV NS1 protein that is recognized by MAb D9. This MAb and its epitope may facilitate future studies on NS1 function and aid the development of new diagnostic methods for AIV detection.

## Background

Avian influenza (AI) is an infectious disease caused by avian influenza virus (AIV), a member of the *Orthomyxoviridae* family [1, 2]. AIV is an enveloped, negative-stranded, segmented RNA virus. The AIV genome consists of eight RNA segments that vary in size ranging from 890 to 2,341 bases and code for 11 known proteins. AIV produces 11 viral proteins that can be divided into three main categories: surface proteins, internal proteins, and nonstructural proteins [3]. The viral particle contains three surface proteins: hemagglutinin (HA), neuraminidase (NA), and matrix 2 (M2) proteins. The internal proteins of the viral particle include three polymerase proteins, PA, PB1, and PB2, as well as nucleoprotein, matrix 1 (M1), and nonstructural protein 2 (NS2). Nonstructural protein 1 (NS1) is a protein that is not packaged into the virus particle, although it is produced in large quantities in infected cells [4, 5]. NS1 protein plays an important role in virus infection and in the viral replication process. It has been shown to counteract the host type I interferon response [6], to mediate host cell apoptosis [7, 8], and to regulate the process of protein synthesis [9].

AIV can be classified into high (HPAIV) and low (LPAIV) pathogenic avian influenza viruses depending on the severity of the diseases that they cause, ranging from asymptomatic infections to acute systemic diseases and even death [10]. During the last decades, HPAIV has been involved in several outbreaks in poultry and wild birds around the world [11]. The epidemic of AI represented by the H5N1 subtype continues to cause severe losses for the poultry industry and threatens both human health and public health worldwide. Vaccination against AIV, despite being a powerful tool for disease control, makes distinguishing between infected and vaccinated animals through serological tests difficult [12]. To overcome this problem, several studies have focused on a differentiation of infected and vaccinated animals program of AI, which is mostly centralized on the NS1 protein-based antibody response [13]. Our study aimed to contribute to this program by generating a monoclonal antibody (MAb) to NS1 and identifying the epitope recognized by this MAb.

## Results

### Expression and characterization of a recombinant AIV NS1 protein

**Fig. 1:**
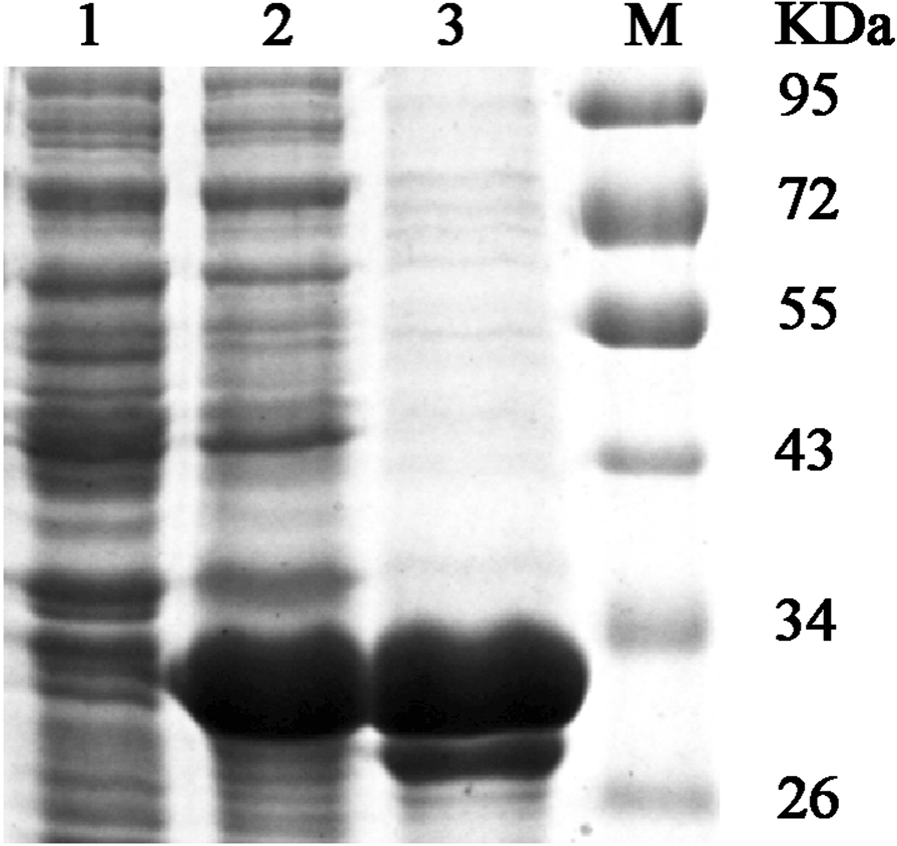
SDS-PAGE analysis of the recombinant NS1 protein and purified NS1 protein. M, Marker. Lane 1, proteins expressed in *E. coli* without IPTG induction. Lane 2, proteins expressed in *E. coli* with IPTG induction. Lane 3, purified recombinant NS1 protein

**Fig. 2:**
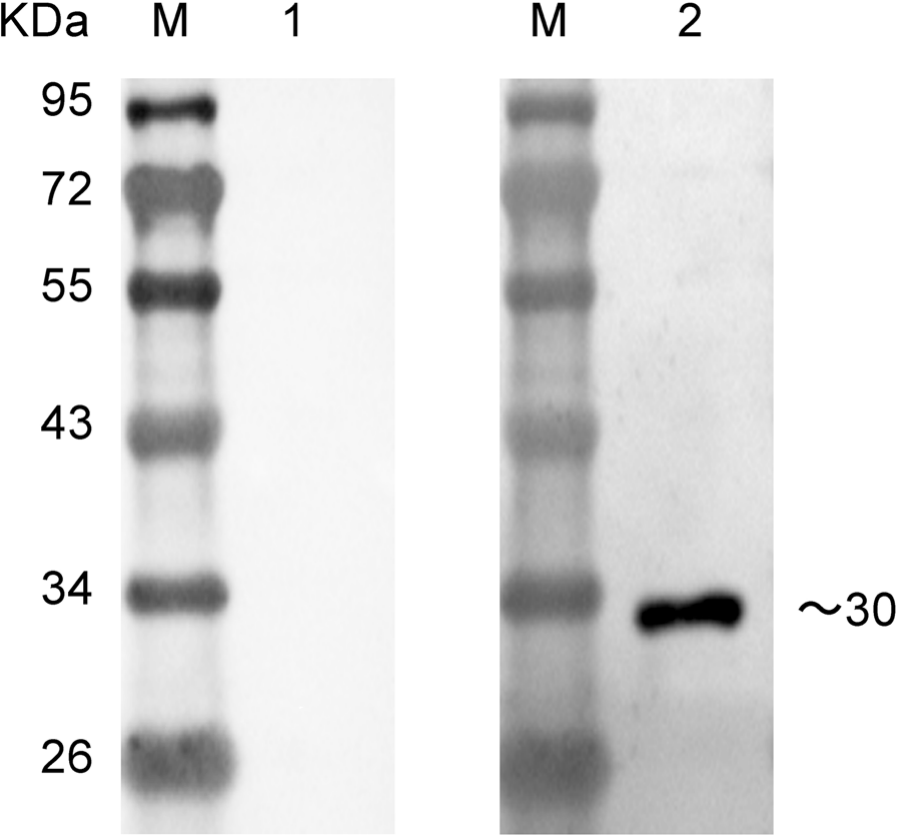
Western blot analysis of purified recombinant NS1 protein. M, Marker. Lane 1, the binding of purified recombinant NS1 protein with AIV-negative chicken sera. Lane 2, the binding of purified recombinant NS1 protein with AIV-positive chicken sera

The recombinant NS1 protein was expressed predominantly as an inclusion body in *E. coli* after IPTG induction. It was purified by affinity chromatography and analyzed by SDS-PAGE (Fig. 1). Western blot analysis indicated that the purified recombinant NS1 protein, which was encoded by the gene from the H5N1 virus, was reactive with AIV-positive sera (Fig. 2). These results show that the purified recombinant NS1 protein was a suitable antigen for immunization and hybridoma screening.

### Production and characterization of NS1-specific MAb

**Fig. 3:**
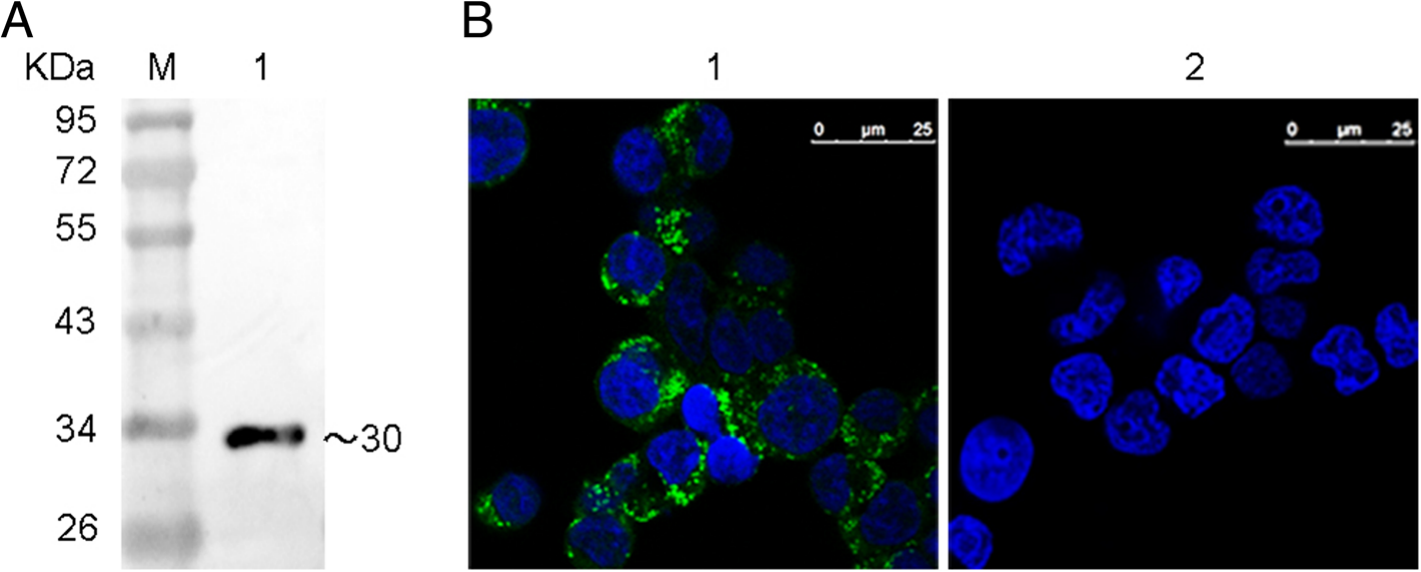
The binding of MAb D9 with prokaryotic and eukaryotic expression of NS1 protein. The binding of MAb D9 with prokaryotic purified recombinant NS1 protein (**a**) and NS1 protein expressed in 293T cells transfected with eukaryotic recombinant plasmids (**b**). 1. The binding of MAb D9 with NS1 protein expressed in 293T cells. 2. The binding of negative serum with NS1 protein expressed in 293T cells

Purified recombinant NS1 protein expressed in *E. coli* was used to immunize BALB/c mice. After cell fusion and screening, several hybridoma cell lines were obtained, which produced NS1-specific MAbs. One MAb produced by the line, designated as D9, was selected for its strong reactivity with recombinant NS1 protein in indirect ELISA (data not shown) and western blot (Fig. 3a) assays. Furthermore, confocal laser scanning microscopy results show that MAb D9 had a strong reactivity with NS1 expressed in 293T cells transfected with eukaryotic recombinant plasmids (Fig. 3b). MAb D9 was composed of an IgG_1_ heavy chain paired with a κ-type light chain, as determined using the SBA Clonotyping™ System/HRP Kit according to the manufacturer’s instructions. The titers of MAb D9 in hybridoma cell culture supernatants and in ascites were measured by indirect ELISA and determined to be 1:1,000 and 1:100,000, respectively.

### Phage enrichment

**Fig. 4:**
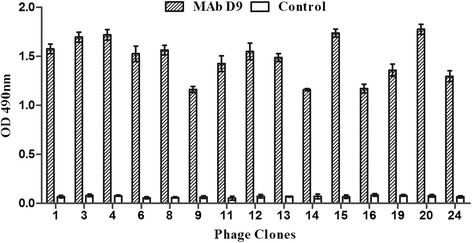
Detection of the selected phages for MAb D9 binding by phage ELISA. After three successive rounds of biopanning, phage ELISA was performed for further identification of the positive phage clones that were successfully sequenced. An irrelevant MAb was used as a negative control. Error bars represent the standard deviation of the results from three replicate experiments

To define the MAb binding epitope, a phage-displayed 7-mer random peptide library was screened with MAb D9. The output to input ratios of the three rounds of biopanning were 2.2 × 10^−4^, 8.5 × 10^−3^, and 3.4 × 10^−2^, respectively, which indicated an enrichment of phages bound to MAb D9. After three successive rounds of biopanning, the selected 24 phage clones showed higher reactivity by phage ELISA with MAb D9 than with a negative control of irrelevant MAb (data only shown for phage clones that were successfully sequenced; Fig. 4).

### Epitope prediction

The selected 24 phage clones were sequenced and analyzed by DNASTAR Lasergene program (Windows version; DNASTAR Inc., Madison, WI, USA). The MAb D9 recognized phages displaying peptides with the consensus peptide WNLNTV--VS, which was substantially matched with ^182^WNDNTVRVS^190^ of AIV NS1 protein (Table 1).

**Table 1:** Sequence comparison of random peptide inserts displayed on the positive phage

Phage clones	Amino acid sequence of the insert									
1	G	**W**	S	L	S	**T**	-	-	**V**	-
3	-	**W**	R	**D**	**N**	A	I	A	-	-
4	-	**W**	**N**	L	**N**	**T**	T	N	-	-
6	-	**W**	**N**	L	**N**	H	I	F	-	-
8	-	**W**	S	Q	T	**T**	-	-	**V**	**S**
9	-	**W**	G	E	**N**	**T**	I	W	-	-
11	-	F	**N**	L	**N**	**T**	A	M	-	-
12	-	**W**	**N**	S	**N**	A	I	-	-	**S**
13	A	**W**	**N**	F	**N**	S	Q	-	-	-
14	-	**W**	**N**	F	-	**T**	A	R	E	-
15	-	**W**	**N**	Q	**N**	**T**	A	-	-	**S**
16	-	**W**	T	Y	T	**T**	F	E	-	
19	-	**W**	S	Q	T	**T**	-	-	**V**	**S**
20	-	**W**	**N**	Q	**N**	**T**	-	-	**V**	**S**
24	-	**W**	**N**	S	**N**	A	I	-	-	**S**
Consensus	-	**W**	**N**	L	**N**	**T**	**V**	-	**V**	**S**
AIV-NS1	E	**W**	**N**	D	**N**	**T**	**V**	R	**V**	**S**

Note: Conservative amino acids are bold

### Epitope identification

**Fig. 5:**
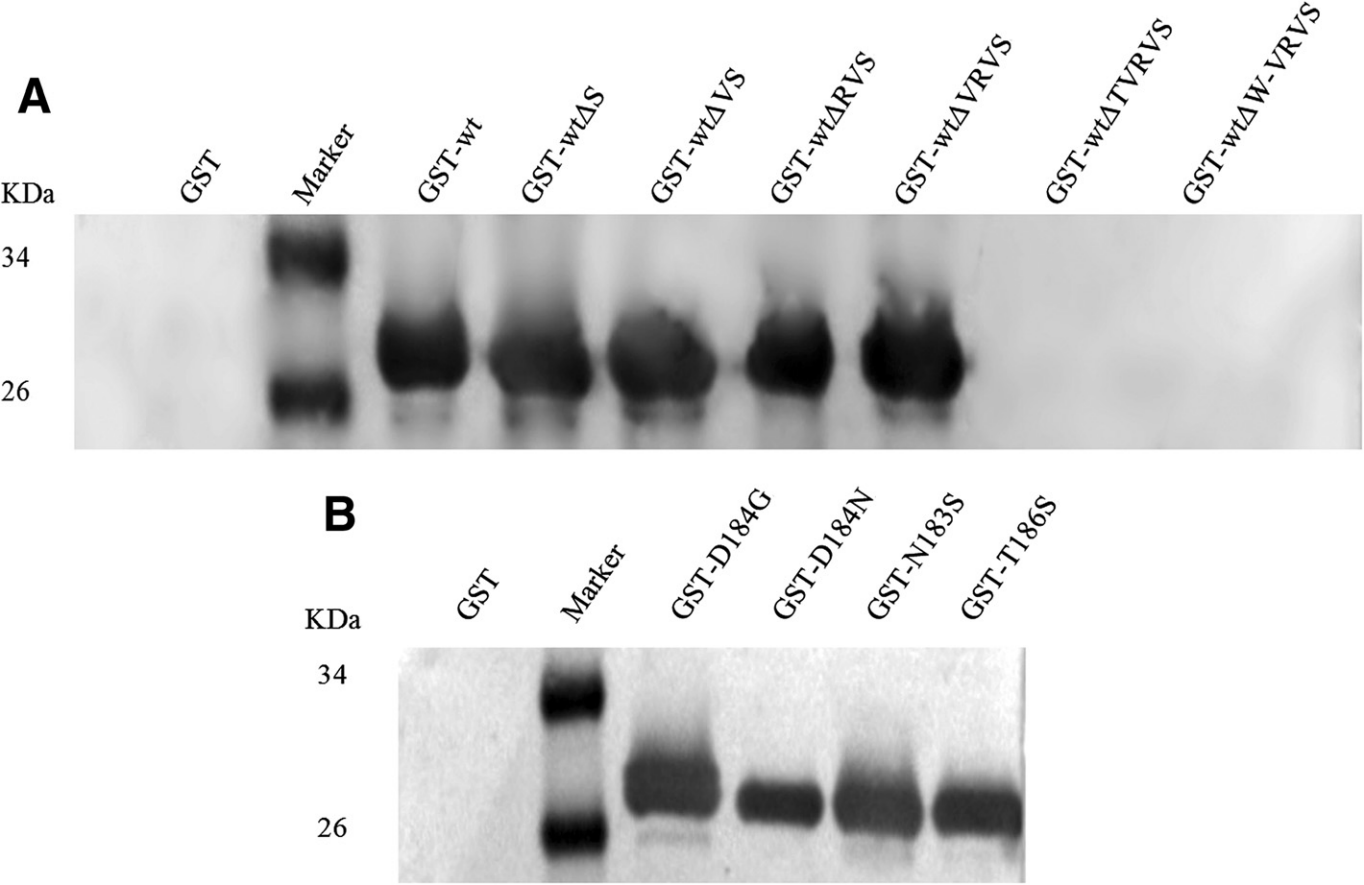
Reactivity of the GST-fusion proteins with the MAb D9. Western blots were performed to test the reactivity of MAb D9 with the GST-fusion proteins containing wild-type and truncated (**a**) or mutant (**b**) motifs derived from the motif WNDNTVRVS. Marker: PageRuler™ Prestained Protein Ladder. GST-7wt: fusion protein containing the motif WNDNTVRVS, GST-wtΔS: WNDNTVRV, GST-wtΔVS: WNDNTVR, GST-wtΔRVS: WNDNTV, GST-wtΔVRVS: WNDNT, GST-wtΔTVRVS: WNDN, GST-wtΔW-VRVS: NDNT, GST-T186S: WNDNS, GST-N183S: WSDNT, GST-D184G: WNGNT and GST-D184N: WNNNT

To confirm the linear epitope recognized by MAb D9, the motif WNDNTVRVS was expressed as glutathione S-transferase (GST) fusion protein GST-wt. Western blot analysis results show that MAb D9 reacted with GST-wt (Fig. 5a). To further identify the minimal epitope, six truncated motifs, GST-wtΔS, GST-wtΔVS, GST-wtΔRVS, GST-wtΔVRVS, GST-wtΔTVRVS, and GST-wtΔW, were expressed in *E. coli* and then subjected to western blot with MAb D9. The results show that except for GST-wtΔTVRVS and GST-wtΔW, the other motifs were recognized by the MAb D9, suggesting that the motif WNDNT represents a minimal unit for the reactivity of the epitope recognized by MAb D9 (Fig. 5a). Taken together, these results show that WNDNT is a minimal linear B-cell epitope of AIV NS1 protein.

**Fig. 6:**
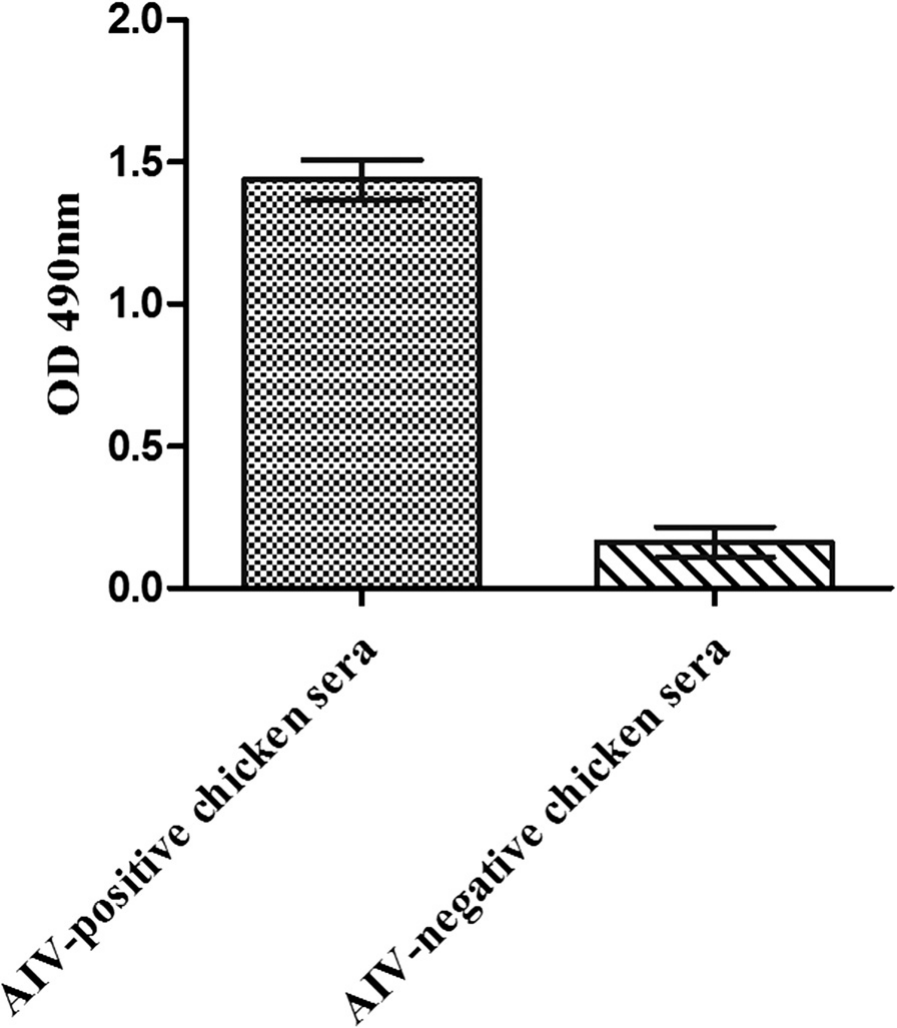
ELISA analysis for reactivity of the identified epitope with AIV-positive chicken sera. Expressed protein (the minimal epitope: GST-wtΔVRVS) was used to coat the 96-well plates. AIV-positive chicken sera was used to detect the coated proteins, AIV-negative chicken sera was included as a negative control. Error bars represent the standard error of the mean of the results from three replicate experiments

### Reactivity of the identified epitope with AIV-positive chicken sera

To verify whether the identified epitope could be recognized by AIV-positive chicken sera, an indirect ELISA assay was performed. 96-well plates were coated with the protein GST-wtΔVRVS. The ELISA results indicated that the identified epitope peptide WNDNT could be recognized by the AIV-positive chicken sera but not by AIV-negative chicken sera, revealing that the epitope exhibited good reactivity and would be useful for the diagnosis of AIV (Fig. 6).

### Homology analysis

All sequences in the NCBI Influenza Virus Sequence Database were aligned using clustal software to evaluate the degree of conservation of the linear epitope recognized by MAb D9 among AIV. The resulting alignment indicated that the epitope ^182^WNDNT^186^ is highly conserved among different isolates, although in some sequences a single amino acid substitution was identified (the first N is replaced by S, the D is replaced by G or N, or the T is replaced by S). Based on these amino acid substitutions, we expressed four mutant motifs, GST-T186S, GST-N183S, GST-D184G, and GST-D184N, as GST fusion proteins. Western blot assays indicated that MAb D9 could recognize all of the fusion proteins (Fig. 5b). The amino acid substitutions at different sites within the epitope among some isolates did not affect the reactivity of MAb D9 with the epitope, which suggested that MAb D9 was broadly recognized by almost all AIV NS1.

## Discussion

As with other viruses, the antigenic structures of AIV are recognized by the immune system through binding of its epitopes by the surface receptors on specific clones of T cells or B cells, inducing a humoral and/or cell-mediated immune response [14]. To our knowledge, previous studies of epitopes against AIV have mainly focused on the HA protein rather than on the NS1 protein [15–17]. Thus, the identification of an AIV epitope on NS1 protein is important for understanding influenza virus pathogenesis. This finding will facilitate further study on the molecular mechanisms responsible for viral stimulation of the immune response.

MAb combined with phage display is one of the most widely used and fastest-growing tools for mapping epitopes [18, 19]. It has been successfully applied to identify a variety of pathogenic microorganism epitopes. Compared with other existing methods, phage display is a simple, fast, and convenient method for mapping epitopes [20]. Moreover, the phage display peptide library can be screened to affinity-select mimotopes, which are peptides that mimic discontinuous epitopes [21]. Screening of a 12-mer peptide library was performed for the identification of a conserved linear B-cell epitope of the E2 glycoprotein N-terminus of classical swine fever virus [22]. Sun et al. similarly screened a 12-mer peptide library using phage-display technology to define the linear epitope recognized by West Nile virus C protein-specific MAb [23]. Here, we used purified recombinant NS1 expressed in *E. coli* to generate MAb D9. Then, by screening a phage display random 7-mer peptide library with MAb D9, the motif WNDNTVRVS located from AA182 to AA190 of the AIV NS1 protein was defined as the linear epitope of MAb D9. Furthermore, by using a set of truncated polypeptides expressed as GST fusion proteins, we identified WNDNT as a minimal linear B-cell epitope of AIV NS1 protein.

To investigate the conservation of the identified linear epitope among AIV viruses, all sequences in the NCBI Influenza Virus Sequence Database were aligned using clustal software. There are 23,072 NS1 protein sequences (6,854 sequences after identical sequences were collapsed) in total in the database. 60.23 % of the sequences contain the epitope WNDNT. 36.44 % of the sequences have a unique amino acid substitution, with 6,061 sequences that replace D with G, 2,265 sequences that replace T with S, 62 sequences that replace D with N, and 19 sequences that replace the first N with S. Amino acid substitution or lack of it was not related to the hosts and subtypes of the viruses. These results show that the epitope is present widely in influenza virus strains (Table 2). Among the mutated amino acids, D, G, and N are polar amino acids and N, S, and T are uncharged polar amino acids.

**Table 2:** Sequence comparison of the identified epitope among AIV strains available in the NCBI Influenza Virus Sequence Database

Different variations	Sequences	Amino acid substitutions				
No substitution	13895	W	N	D	N	T
Single substitution	6061	-	-	G	-	-
	2265	-	-	-	-	S
	62	-	-	N	-	
	19	-	S	-	-	-
The rest	770					
Total	23072					

Many epitopes have been defined in the NS1 protein of AIV viruses, and most of them have been mapped by MAbs produced in mice immunized with recombinant proteins or synthesized peptides. Among the previously-mapped epitopes, there are seven epitopes that do not overlap with the epitope identified in this study. These epitopes are LEWNDNTVRVTETIQRFAWRNSDEDGRLPLPPNQKR [24], EWNDNTVRVSETLQRFA [25, 26], VLIGGLEWNDNTVRVSE [27–29], EWNDNTVRVSETLQR and LIGGLEWNDNTVRVS [30], GLEWNDNTV [31], and EWNDNTVRV [32]. There are also three epitopes that contain part of the epitope identified in this study. They are VKNAIGVLIGGLEWNDN [29], DEDVKNAIGVLIGGLEWNDN [33], and NAVGVLIGGLEWNDN [30]. Because none of these epitopes are completely identical to the epitope described in this study, this new epitope is another different linear epitope on the AIV NS1 protein. The MAb and its epitope described in this study will facilitate further study on the function of NS1 and aid in diagnostic method development for AIV detection.

## Conclusions

This study identified the motif WNDNT as a conserved linear epitope on the AIV NS1 protein that is recognized by the MAb D9. This MAb and its epitope may facilitate further study on the pathogenesis of AIV and aid in diagnostic method development for AIV detection.

## Methods

### Ethics statement

Care of laboratory animals and animal experimentation were performed in accordance with animal ethics guidelines and approved protocols. All animal studies were approved by the Review Board of Harbin Veterinary Research Institute of the Chinese Academy of Agricultural Sciences and by the Animal Care and Use Committee of Heilongjiang Province (SYXK (Hei) 2012–2067).

### Plasmid and cells

The recombinant plasmid pET-NS1 containing the AIV NS1 gene sequence was generated in our lab. The myeloma cells SP2/0 and 293T cells were both cultured in Dulbecco’s modified Eagle’s medium (DMEM, GIBCO, Grand Island, NY, USA) in a humidified 5 % CO_2_ atmosphere at 37 °C. All culture media were supplemented with 10 % heat-inactivated fetal bovine serum (Hyclone, Thermo, Rockford, IL, USA) and antibiotics (10,000 μg/mL of streptomycin and 10,000 units/mL of penicillin, GIBCO).

### Prokaryotic expression and purification of recombinant AIV NS1 protein

The pET-NS1 was transformed into *E. coli* BL21 (DE3), and transformed bacteria were identified using both restriction enzyme digestion and PCR. Further confirmation was obtained by DNA sequencing. DE3 transformed with pET-NS1 were cultured at 37 °C in culture medium (per liter: 10 g tryptone, 5 g yeast extract, and 10 g NaCl, adjusted pH to 7.0) supplemented with kanamycin (100 μg/μL). After several hours of shaking, when the optical density (OD 600 nm) was up to 0.6, isopropyl-D-1-thiogalactopyranoside (IPTG, Takara, Dalian, China) was added to the medium at a final concentration of 1 mM for the induction of protein expression. Then, an additional 4 h incubation at 37 °C with agitation was performed.

After the cells were harvested by centrifugation at 10,000 × g for 10 min at 4 °C, the pelleted bacteria were suspended in 10 mL phosphate-buffered saline (PBS) and then lysed by sonication in an ice water bath. The pellet was re-suspended with 10 mL of 1 × Binding Buffer from the His-band purification kit (Novagen, Merck KGaA, Darmstadt, Germany) plus 6 M urea. After an ice water bath, the suspension was centrifuged at 16,000 × g for 30 min. The supernatant was filtered by a 0.45-μm filter membrane. The His-band purification kit was used according to the manufacturer’s instructions for purification of the NS1 protein. After Ni-charged binding, washing, and elution, the purified protein was ultrafiltered to fold its structure. The purified protein was analyzed in SDS-polyacrylamide gel electrophoresis (SDS-PAGE) and visualized using Coomassie brilliant blue staining.

### Western blots

Western blots were carried out to analyze the antigenicity of the purified protein. After being separated by 10 % SDS-PAGE, the purified protein was electrically transferred onto a polyvinylidenefluoride (PVDF) membrane. Then, the membrane was blocked with 5 % skim milk in PBS for 1 h at 37 °C, followed by incubation with AIV-positive sera diluted in 5 % skim milk in PBS for 1 h at 37 °C. After three washes with PBST (PBS containing 0.05 % Tween-20), the membrane was incubated with a 1:4,000 dilution of horseradish peroxidase (HRP)-conjugated goat anti-chicken IgG (Sigma, St. Louis, MO, USA) for 1 h at 37 °C. Color was developed with 3,3′-diaminobenzidinetetrahydrochloride (DAB) and the reaction was stopped by rinsing with distilled water and drying the membrane.

### Production and identification of MAb against AIV NS1 protein

To prepare MAb against AIV NS1, six-week-old BALB/c mice were intraperitoneally immunized with 100 μg purified recombinant NS1 protein expressed in *E. coli* emulsified with an equal volume of Freund’s complete adjuvant (Sigma) and boosted with the same dose 2 weeks later. Three days after the final booster, the spleen cells from the immunized mice were fused with myeloma cells SP2/0, and hybridoma supernatants were screened by indirect ELISA as described below. Cells whose supernatants produced positive results in the ELISA were subcloned by limiting dilution and further characterized by western blot (described above) and confocal laser scanning microscopy.

### Indirect ELISA

96-well plates were sensitized with 100 μL/well of purified recombinant NS1 protein diluted in carbonate-bicarbonate buffer (pH 9.6) at 4 °C overnight. After being blocked with 5 % skim milk in PBS for 1 h at 37 °C, the plates were incubated with hybridoma supernatants for 1 h at 37 °C. Then, the plates were incubated with a 1:40,000 dilution of HRP-conjugated goat anti-mouse IgG (Sigma) for 1 h at 37 °C. A substrate solution containing o-phenylenediamine (OPD) was added for color development. Following a 15 min incubation, the enzymatic reaction was stopped by the addition of 100 μL/well of 2 M H_2_SO_4_ and the absorbance was measured at 490 nm using a Microplate Reader (iMark, Bio-Rad Laboratories, Hercules, CA).

### Confocal laser scanning microscopy

When 293T cells were approximately 70–80 % confluent, the cells were transfected with eukaryotic recombinant plasmids pCAGGs-NS1 by using transfection reagent (Invitrogen, Carlsbad, CA, USA) according to the manufacturer’s instructions. Thirty-six hours later, the cells were fixed with 4 % polyoxymethylene in PBS overnight at 4 °C. Then, 0.5 % Triton-X 100 in PBS was added to permeabilize cells for 20 min at room temperature. The microplates were blocked with 5 % skim milk in PBS for 30 min at room temperature. After being incubated with hybridoma supernatants for 1 h at 37 °C, the microplates were incubated with fluorescein isothiocyanate (FITC)-conjugated goat anti-mouse IgG (Santa Cruz Biotechnology, Dallas, TX, USA) diluted in PBS containing 0.5 % Tween-20 for 1 h at 37 °C, followed by DAPI (Sigma) diluted in PBS for 5 min at 37 °C. After being rinsed three times with PBS and mounted in PBS with 10 % glycerol, the microplates were viewed under a fluorescence microscope (TCS SP5, Leica Microsystems GmbH, Wetzlar, Germany).

### Biopanning

A Ph.D.-7™ Phage Display Peptide Library Kit was purchased from New England Biolabs Inc (Hitchin, Herts, UK). Biopanning was done according to the manufacturer’s instructions. Briefly, biopanning was carried out in 96-well microtiter plates. One well was coated with 100 μg/mL MAb in 0.1 M NaHCO_3_ (pH 8.6) overnight at 4 °C with gentle agitation in a humidified container. Then, the well was blocked with blocking buffer (0.1 M NaHCO_3_, pH 8.6, containing 5 mg/mL BSA) at 4 °C for 2 h. The original phage library diluted in TBST (50 mM Tris–HCl, pH 7.5, 150 mM NaCl plus 0.1 % Tween-20), containing 4 × 10^10^ pfu phage clones, was pipetted onto the coated well and rocked gently for 1 h at room temperature. The unbound phages were washed away, and the specifically-bound phages were eluted with 0.2 M glycine-HCl (pH 2.2, containing 1 mg/mL BSA) and were immediately neutralized with 1 M Tris–HCl (pH 9.1). Approximately 1 μL of the eluate was titered on LB/IPTG/Xgal plates for calculating the ratio of output to input, and the rest was amplified by adding it to ER2738 culture and incubating the culture for 4.5 h at 37 °C with vigorous shaking. The amplified eluates were finally titered on LB/IPTG/Xgal plates for calculating an input volume in the following rounds. In the two successive rounds of biopanning, the MAb concentration was reduced to 75 μg/mL and 50 μg/mL and the Tween-20 concentration in the wash steps was raised to 0.3 % and 0.5 %, respectively. After three rounds of biopanning, blue plaques were stabbed from one of titering plates and amplified in a tube containing ER2738 culture for use in a phage ELISA and for sequencing as described below.

### Phage ELISA

96-well plates were coated with 150 μL of 100 μg/mL MAb in 0.1 M NaHCO_3_, pH 8.6, overnight at 4 °C. The plates were completely filled with blocking buffer (0.1 M NaHCO_3_, pH 8.6, containing 5 mg/mL BSA) at 4 °C for 2 h. Additionally, uncoated wells for each clone were also blocked as negative controls. A second, fully uncoated microtiter plate was blocked for use in serial dilutions of phages before their addition to the coated wells. Fourfold serial dilutions of the phage were carried out in the separate blocked plate, starting with 10^12^ virions and ending with 2 × 10^5^ virions. Then, the dilutions were transferred to both the coated and uncoated wells and the plates were incubated at room temperature for 1 h with agitation. HRP-conjugated anti-M13 monoclonal antibody (GE Healthcare, Piscataway, NJ, USA) was used as the secondary antibody at a 1:4,000 dilution in blocking buffer at 37 °C for 1 h, followed by color development with the substrate solution OPD. The reaction was stopped after 15 min by adding 100 μL of 2 M H_2_SO_4_ to each well, and then the absorbance was measured at 490 nm on a Microplate Reader (iMark, Bio-Rad Laboratories).

### Sequencing of DNA displayed on the positive phages

Sequencing templates were purified using a purification kit as described in the manufacturer’s instructions (New England Biolabs). The DNA inserts were sequenced with the -96gIII sequencing primer: 5′-CCCTCATAGTTAGCGTAACG-3′. The amino acid sequences deduced from the inserted nucleotide sequences were analyzed by the DNASTAR Lasergene program.

### Precise localization of the epitope

To verify if the identified motif represented an epitope recognized by MAb D9, a series of complementary oligonucleotides encoding for wild-type and truncated versions of the motif WNDNTVRVS were synthesized, annealed, and cloned into the *Bam*HI/*Xho*I sites of the pGEX-6p-1 vector, producing a group of recombinant plasmids. After DNA sequencing, the recombinant proteins were expressed in *E. coli* BL21 (DE3) competent cells with the induction of 0.8 mM IPTG. Eight segments with truncations at the amino- or carboxy-termini of the motif WNDNTVRVS were constructed to express the GST fusions GST-wildtype (GST-wt), GST-wtΔS, GST-wtΔVS, GST-wtΔRVS, GST-wtΔVRVS, GST-wtΔTVRVS, and GST-wtΔW-VRVS representing WNDNTVRVS, WNDNTVRV, WNDNTVR, WNDNTV, WNDNT, WNDN, and NDNT, respectively (Table 3). Western blots were carried out as described above to identify the reactivity with these polypeptides with MAb D9 to determine the minimal unit of the epitope.

**Table 3:** The oligonucleotides coding for wild type and truncated or mutant motifs

Name	The sequences of oligonucleotides	Coding motifs
GST-wt-F	5'-*GATCC*TGGAATGATAACACAGTTCGAGTCTCT**TAA** *C*-3'	WNDNTVRVS
GST-wt-R	5'-*TCGAG* **TTA**AGAGACTCGAACTGTGTTATCATTCCA*G*-3'	
GST-wtΔS-F	5'-*GATCC*TGGAATGATAACACAGTTCGAGTC**TAA** *C*-3'	WNDNTVRV
GST-wtΔS-R	5'-*TCGAG* **TTA**GACTCGAACTGTGTTATCATTCCA*G*-3'	
GST-wtΔVS-F	5'-*GATCC*TGGAATGATAACACAGTTCGA**TAA** *C*-3'	WNDNTVR
GST-wtΔVS-R	5'-*TCGAG* **TTA**TCGAACTGTGTTATCATTCCA*G*-3'	
GST-wtΔRVS-F	5'-*GATCC*TGGAATGATAACACAGTT**TAA** *C*-3'	WNDNTV
GST-wtΔRVS-R	5'-*TCGAG* **TTA**AACTGTGTTATCATTCCA*G*-3'	
GST-wtΔVRVS-F	5'-*GATCC*TGGAATGATAACACA**TAA** *C*-3'	WNDNT
GST-wtΔVRVS-R	5'-*TCGAG* **TTA**TGTGTTATCATTCCA*G*-3'	
GST-wtΔTVRVS-F	5'-*GATCC*TGGAATGATAAC**TAA** *C*-3'	WNDN
GST-wtΔTVRVS-R	5'-*TCGAG* **TTA**GTTATCATTCCA*G*-3'	
GST-wtΔW-VRVS-F	5'-*GATCC*AATGATAACACA**TAA** *C*-3'	NDNT
GST-wtΔW-VRVS-R	5'-*TCGAG* **TTA**TGTGTTATCATT*G*-3'	
GST-N183S-F	5'-*GATCC*TGGTCTGATAACACA**TAA** *C*-3'	WSDNT
GST-N183S-R	5'-*TCGAG* **TTA**TGTGTTATCAGACCA*G*-3'	
GST-D184G-F	5'-*GATCC*TGGAATGGAAACACA**TAA** *C*-3'	WDGNT
GST-D184G-R	5'-*TCGAG* **TTA**TGTGTTTCCATTCCA*G*-3'	
GST-D184N-F	5'-*GATCC*TGGAATAATAACACA**TAA** *C-*3'	WNNNT
GST-D184N-R	5'-*TCGAG* **TTA**TGTGTTATTATTCCA*G-*3'	
GST-T186S-F	5'-*GATCC*TGGAATGATAACTCA**TAA** *C*-3'	WNDNS
GST-T186S-R	5'-*TCGAG* **TTA**TGAGTTATCATTCCA*G*-3'	

Note: All primers expressing GST fusion proteins carried the *Bam*HI restriction endonuclease site upstream and the *Xho*I site downstream, which are shown in italics. The stop codons are shown in bold

### Detection of the reactivity of the identified epitope with AIV-positive chicken sera

To determine whether the identified epitope could be recognized by AIV-positive chicken sera, 96-well plates were coated with the minimal epitope WNDNT (5 μg/mL, 100 μL/well) diluted with the carbonate-bicarbonate buffer (pH 9.6) at 4 °C overnight and then blocked with 200 μL/well of 5 % skim milk diluted by PBS. After three washes with PBST, AIV-positive chicken sera and AIV-negative chicken sera (negative control) were added to the plates at a 1:200 dilution and were incubated at 37 °C for 1 h. Next, 100 μL of anti-chicken IgY HRP- conjugate (Sigma) at a 1:10000 dilution was added to the plates after three washes with PBST and incubated at 37 °C for 1 h. The plates were then washed three times and 100 μL/well of OPD was added. After 15 min of incubation at room temperature, the reaction was stopped by the addition of 100 μL/well of 2 M H_2_SO_4_ and the absorbance was measured at 490 nm using a Microplate Reader (iMark, Bio-Rad Laboratories).

### Homology analysis

To investigate the conservation of the identified linear epitope among AIV viruses, all sequences in the NCBI Influenza Virus Sequence Database (http://www.ncbi.nlm.nih.gov/genomes/FLU/FLU.html) were aligned using clustal software. Based on the results of the alignment, four mutant motifs were expressed as GST fusion proteins (designated as GST-T186S, GST-N183S, GST-D184G, and GST-D184N representing WNDNS, WSDNT, WNGNT, and WNNNT) as above (Table 3) to investigate whether amino acid substitutions at different sites affect the ability of MAb D9 to react with the epitope.
